# Prevalence of shrimp allergy: a meta-analysis based on different diagnostic methods

**DOI:** 10.3389/falgy.2025.1635274

**Published:** 2025-09-01

**Authors:** Jiaqi Chen, Qiang Zhang, Yongli Ying, Xuying Zhang, Chunsheng Qu

**Affiliations:** 1 Clinical Laboratory of the Lishui Hospital of Wenzhou Medical University, The First Affiliated Hospital of Lishui University, Lishui People’s Hospital, Lishui, Zhejiang, China; 2 Nursing Department of the Lishui Hospital of Wenzhou Medical University, The First Affiliated Hospital of Lishui University, Lishui People’s Hospital, Lishui, Zhejiang, China

**Keywords:** shrimp allergy, prevalence, sensitization, meta-analysis, food allergy epidemiology

## Abstract

**Background:**

Shrimp allergy (SA) represents a significant public health concern, yet its overall prevalence remains unclear.

**Method:**

A systematic search of PubMed, Web of Science, and Embase through September 30, 2024 identified 40 studies that reported SA prevalence using self-reported symptoms, physician diagnosis, skin prick tests, specific IgE, or food challenge tests.

**Results:**

The pooled prevalence was estimated at 1.90% for self-reported symptomatic SA and 1.94% for self-reported physician-diagnosed SA, while testing via skin prick or specific IgE yielded a prevalence of 2.76%. Notably, symptomatic testing showed a lower prevalence of 0.43%, and food challenge tests confirmed a prevalence of 0.50%. Considerable heterogeneity was observed across studies, with prevalence varying by region and age group, and no publication bias was detected.

**Conclusion:**

These findings indicate that the prevalence of SA varies with diagnostic criteria, age, and region, underscoring the need for harmonized diagnostic standards to improve prevalence estimates and guide public health strategies.

**Systematic Review Registration:**

identifier [CRD420251003956].

## 1 Introduction

In recent years, food allergies have emerged as a growing global public health concern, with both prevalence and associated burden increasing steadily (1). Among various food allergies, shellfish allergy—particularly SA—deserves special attention, given the widespread consumption and affordability of shrimp.

Despite heightened awareness of SA, the pooled prevalence from large-scale studies remains unclear due to variations in study design and diagnostic criteria. These discrepancies largely arise from differences in self-reporting, skin prick tests (SPT), specific IgE (sIgE) measurements, and oral food challenge tests. Methodological inconsistencies across studies have led to substantial variability in prevalence estimates (2–6)^.^ Furthermore, demographic factors such as age and geographic location may influence the prevalence of SA; however, systematic assessments of these factors are currently limited (7).

To address these gaps, a comprehensive meta-analysis is necessary to synthesize existing epidemiological data and provide a more accurate estimate of SA prevalence. The findings of this analysis will offer updated evidence to inform clinical practice and guide public health strategies.

## 2 Methods

### 2.1 Study design and registration

This meta-analysis adhered to the PRISMA (Preferred Reporting Items for Systematic Reviews and Meta-Analyses) guidelines. The study protocol was registered in the PROSPERO database (Registration No.: CRD420251003956).

### 2.2 Search strategy

A comprehensive literature search was conducted in PubMed, Web of Science, and Embase from their inception until September 30, 2024. Considering that in some regions “Shrimp” and “Prawn” are used interchangeably while in others they refer to distinct species, both terms were included. The search combined allergy-related terms (“Allergy”, “Hypersensitivity”, “Anaphylaxis”) with prevalence-related terms (“Prevalence”, “Incidence”, “Epidemiology”, “Risk factors”) using Boolean operators. The search was limited to studies on humans published in English.

In addition to the articles retrieved from the above databases, we also included studies mentioned in the reference lists of relevant articles. These studies were identified from known review articles and reference lists. To ensure the quality and scientific validity of the included studies, we primarily relied on research published in peer-reviewed journals. Therefore, we decided not to include gray literature in this study.

### 2.3 Inclusion and exclusion criteria

Two independent reviewers (Jiaqi Chen and Qiang Zhang) screened the retrieved studies according to predefined criteria, with disagreements resolved through discussion or consultation with a third reviewer (Yongli Ying). The inclusion criteria were as follows: 1. Study Design: Cross-sectional, cohort, or case-control studies. 2. Population: Individuals of all ages with reported SA prevalence. 3. Outcomes: Prevalence data based on one or more of the following: Self-reported symptoms, Physician diagnosis, Skin prick test (SPT), Serum specific IgE (sIgE), Food challenge tests. The exclusion criteria were: 1. Review articles, case reports, conference abstracts, and editorials. 2. Studies lacking relevant prevalence data. 3. Studies using duplicate datasets (only the most comprehensive data was included). 4. Studies that do not meet the definitions or criteria for skin prick test (SPT) or serum specific IgE (sIgE) diagnosis.

### 2.4 Definition of SA

In this study, we adopted a comprehensive approach to identifying SA without distinguishing between IgE-mediated and non-IgE-mediated reactions or differentiating between food allergies and food intolerances. SA was defined based on the following criteria: 1. self-reported symptomatic SA: Individuals experiencing allergic symptoms after shrimp consumption. Referring to an individual experiencing allergic symptoms after shrimp consumption. 2. Self-reported physician-diagnosed SA: SA diagnosed by a physician as reported by the individual. 3. SPT- or sIgE-based SA: Elevated specific IgE levels (≥0.35 kU/L) or a positive skin prick test (SPT) result (wheal ≥ 3 mm larger than the control). 4. SPT- or sIgE-based symptomatic SA: Positive SPT or sIgE results accompanied by allergic symptoms. and 5. Food challenge-confirmed SA: SA established through open, single-blind, or double-blind placebo-controlled food challenge tests.

### 2.5 Data extraction and quality assessment

Two independent reviewers (Jiaqi Chen and Xuying Zhang) extracted data using a pre-designed data extraction form, including study characteristics (first author, publication year, country, study design, sample size, and age group) and outcome measures (prevalence rates and diagnostic methods).

Quality assessment was performed using different appraisal tools based on study design. For cross-sectional studies, the Joanna Briggs Institute (JBI) Critical Appraisal Checklist was used, with studies scoring ≥6 considered high quality, 4–5 as moderate quality, and ≤3 as low quality. For cohort studies, the Newcastle-Ottawa Scale (NOS) was applied, with studies scoring ≥7 classified as high quality, 4–6 as moderate quality, and ≤3 as low quality. Disagreements between reviewers were resolved through discussion.

### 2.6 Statistical analysis

Meta-analysis was conducted using Comprehensive Meta-Analysis (CMA) software. Heterogeneity was assessed using Cochran's *Q* test and the I² statistic, with I² > 50% indicating substantial heterogeneity. Random-effects models were applied to pool the prevalence of SA in the presence of high heterogeneity, while fixed-effects models were used for studies with low heterogeneity (I² < 50% and *P* > 0.05). Publication bias was evaluated using Egger's regression test (8) and Funnel plot. A *P*-value of <0.05 was considered statistically significant for all tests.

For prevalence estimates, we did not differentiate between lifetime and point prevalence. If a study reported both, we used lifetime prevalence for analysis. We prioritized extracting raw data from studies whenever possible; if raw data were unavailable, weighted data were used instead. To estimate the true prevalence of SA, we adjusted for participant dropout in some studies based on the proportion of individuals who completed the study. If a study reported both shrimp and prawn allergy prevalence, we included the data with the larger sample size.

To analyze age-based differences, we categorized study participants as children (<18 years old) or adults (≥18 years old). For studies that included both age groups, classification was based on the predominant age distribution within the study.

## 3 Results

### 3.1 Study selection and characteristics

**Figure 1:**
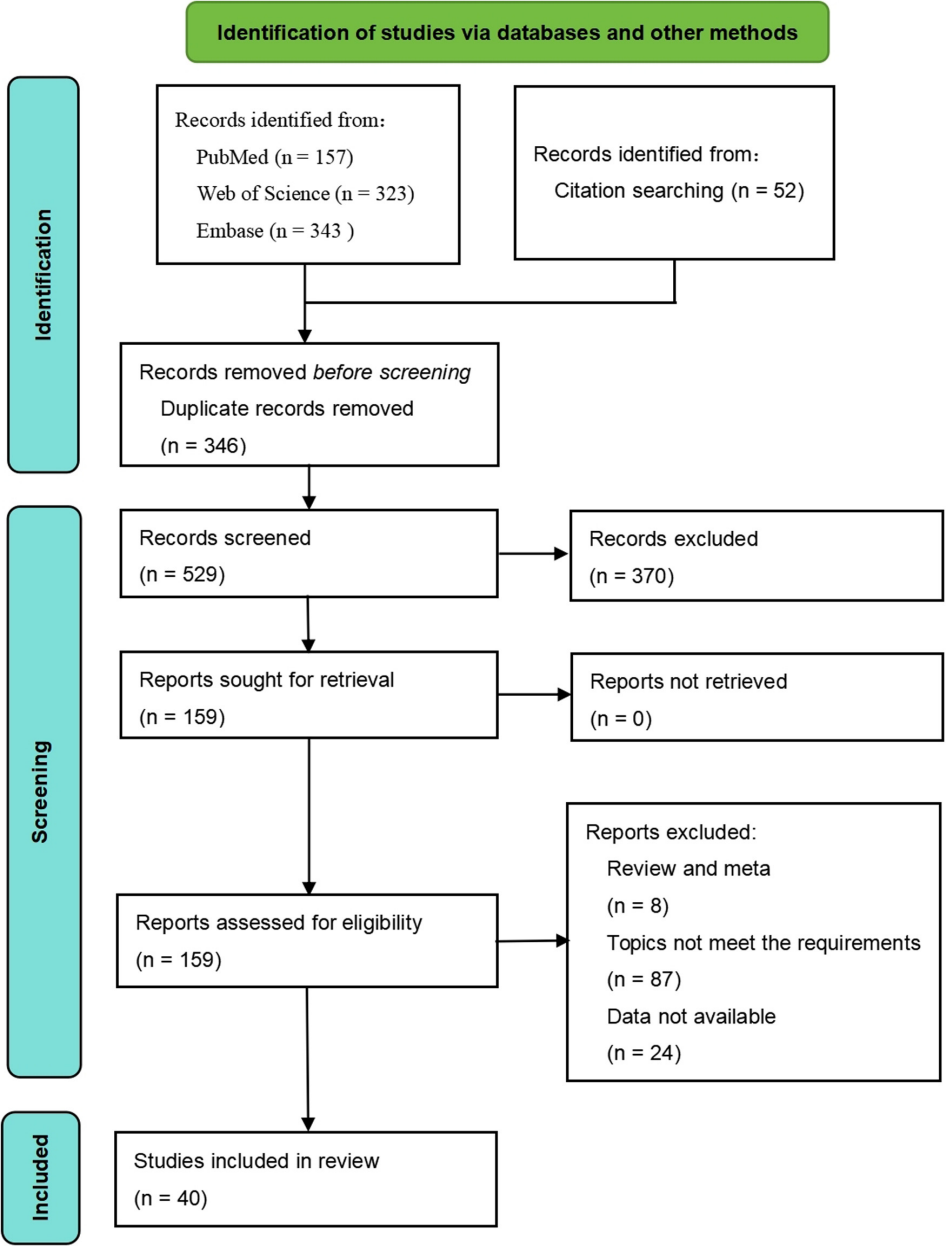
Flow diagram for selection of studies.

A total of 823 articles were identified through the search process, of which 40 studies were ultimately included in the systematic review. The detailed selection process is shown in Figure 1. Among these studies, 36 adopted a cross-sectional design, while 4 employed a cohort design. Regarding study populations, 23 studies focused on children, 12 on adults, and 5 included multiple age groups. The quality assessment revealed that 9 studies were of high quality, 17 were of moderate quality, and 14 were of low quality (Table 1).

**Table 1:** Summary of the characteristics of studies in the meta-analysis.

Author	Year	Country	Region	Study design	Population	Diagnosis	Prevalence (%)	Sample number	Study quality
Beltrán-Cárdenas CE (9)	2021	Colombia	America	Cross- sectional study	Children	SR-SA	1.34	969	Low
Lyons SA (10)	2019	Mixed	Europe	Cross- sectional study	Adult	SR-SA	3.50	17,295	High
						SPT/sIgE-S-SA	0.42	23,405	
Cabrera-Chávez F (11)	2018	ElSalvador	America	Cross- sectional study	Children	SR-SA	1.18	508	Moderate
Ju SY (12)	2015	Korea	Asia	Cross- sectional study	Adult-majority	SR-SA	1.84	543	Low
						PD-SA	1.66	543	
Li J (13)	2020	China	Asia	Cross- sectional study	Children	SPT/sIgE-SA	2.64	16,875	Moderate
Ma Z (14)	2021	China	Asia	Cross- sectional study	Children	SPT/sIgE-S-SA	0.08	1,228	Moderate
Morillo-Argudo DA (4)	2020	Ecuador	America	Cross- sectional study	Children	SR-SA	1.94	1,338	High
						PD-SA	0.75	1,338	
						SPT/sIgE-SA	4.63	1,338	
Ontiveros N (15)	2016	Mexico	America	Cross- sectional study	Children	SR-SA	1.33	1,049	Low
Rangkakulnuwat P (6)	2021	Thailand	Asia	Cross- sectional study	Children	SR-SA	2.85	561	High
						SPT/sIgE-S-SA	1.45	561	
						FC-SA	0.58	344	
Warren CM (16)	2023	USA	America	Cross- sectional study	Children	SR-SA	1.19	38,408	Moderate
					Adult	SR-SA	2.93	40,443	
Zeng GQ (17)	2015	China	Asia	Cross- sectional study	Children	PD-SA	4.41	2,540	Moderate
Woods RK (18)	2002	Australia	Australia	Cross- sectional study	Adult	SPT/sIgE-SA	2.54	1,141	Moderate
						SPT/sIgE-S-SA	0.53	1,141	
Caffarelli C (19)	2011	Italy	Europe	Cross- sectional study	Children	PD-SA	0.00	625	Low
Kjaer HF (20)	2008	Denmark	Europe	Birth cohort study	Children	SPT/sIgE-S-SA	0.00	351	High
Kvenshagen B (21)	2009	Norway	Europe	Birth cohort study	Children	PD-SA	0.00	512	Moderate
Le TM (3)	2015	Holland	Europe	Cross- sectional study	Adult	SR-SA	0.93	3,864	High
						SPT/sIgE-S-SA	0.70	1,430	
						FC-SA	0.00	1,134	
Kavaliunas A (22)	2012	Lithuania	Europe	Cross- sectional study	Children	SR-SA	0.10	3,084	Low
						SPT/sIgE-S-SA	0.03	3,084	
Mossakowska M (23)	2008	Poland	Europe	Cross- sectional study	Adult	SR-SA	0.00	301	Low
Branum AM (24)	2009	USA	America	Cross- sectional study	Children	SPT/sIgE-SA	5.20	3,712	Low
Burney P (25)	2010	Mixed	Mixed	Cross- sectional study	Adult	SPT/sIgE-SA	5.40	4,220	Low
Chen J (26)	2011	China	Asia	Cross- sectional study	Children	SPT/sIgE-SA	0.21	477	High
						FC-SA	0.00	477	
Eggesbo M (27)	1999	Norway	Europe	Birth cohort study	Children	SR-SA	0.50	2,803	Low
Hu Y (28)	2010	China	Asia	Cross- sectional study	Children	SPT/sIgE-SA	0.15	686	Moderate
Lao-araya M (6)	2012	Thailand	Asia	Cross- sectional study	Children	SR-SA	3.36	466	High
						SPT/sIgE-S-SA	3.14	466	
						FC-SA	0.90	466	
Liu AH (29)	2010	USA	America	Cross- sectional study	Children-majority	SPT/sIgE-SA	6.10	2,869	High
					Adult	SPT/sIgE-SA	5.79	4,425	
Obeng BB (30)	2011	Ghana	Africa	Cross- sectional study	Children	SR-SA	0.14	1,407	High
Osterballe M (2)	2005	Denmark	Europe	cohort study	Children-majority	FC-SA	0.11	898	Moderate
					Adult	FC-SA	0.32	936	
Osterballe M (31)	2009	Denmark	Europe	Cross- sectional study	Adult-majority	SR-SA	2.02	843	Low
						FC-SA	1.34	149	
Rance F (32)	2005	France	Europe	Cross- sectional study	Children	SPT/sIgE-SA	0.48	2,716	Low
Santadusit S (5)	2005	Thailand	Asia	Cross- sectional study	Children	SR-SA	1.22	656	Moderate
						SPT/sIgE-S-SA	0.75	268	
						FC-SA	0.75	268	
Wu TC (33)	2012	Taiwan	Asia	Cross- sectional study	Children	PD-SA	3.86	15,982	Low
					Adult	PD-SA	3.27	14,036	
Su KW (34)	2023	Taiwan	Asia	Cross- sectional study	Children	PD-SA	4.25	6,510	Moderate
					Adult	PD-SA	6.12	9,690	
Bedolla-Barajas M (35)	2015	Mexico	America	Cross- sectional study	Adult	SR-SA	4.00	1,126	Moderate
						PD-SA	3.82	1,126	
Mejrhit N (36)	2019	Morocco	Africa	Cross- sectional study	Children	SR-SA	2.07	2,802	Moderate
Domínguez-García MV (37)	2018	America	America	Cross- sectional study	Adult	SR-SA	2.75	1,200	Low
						PD-SA	1.67	1,200	
Gupta RS (38)	2018	USA	America	Cross- sectional study	Children	SR-SA	1.00	41,341	Moderate
						PD-SA	0.57	41,341	
Feng H (39)	2022	China	Asia	Cross- sectional study	Adult	SR-SA	5.79	2,313	Moderate
da S Correia JA (40)	2022	Brazil	America	Cross- sectional study	Children	SR-SA	3.64	412	Low
Xiao S (41)	2023	USA	America	Cross- sectional study	Adult-majority	SR-SA	2.19	6,949	Moderate
Trinh THK (42)	2024	Vietnam	Asia	Cross- sectional study	Adult	SR-SA	11.08	1,038	Moderate

SR-SA, self-reported symptomatic allergy; PD-SA, self-reported physician-diagnosed allergy; SPT/sIgE-SA, SPT- or sIgE-based allergy; SPT/sIgE-S-SA, SPT- or sIgE-based symptomatic allergy; FC-SA, food challenge-confirmed allergy.

### 3.2 Prevalence of self-reported symptomatic SA

**Figure 2:**
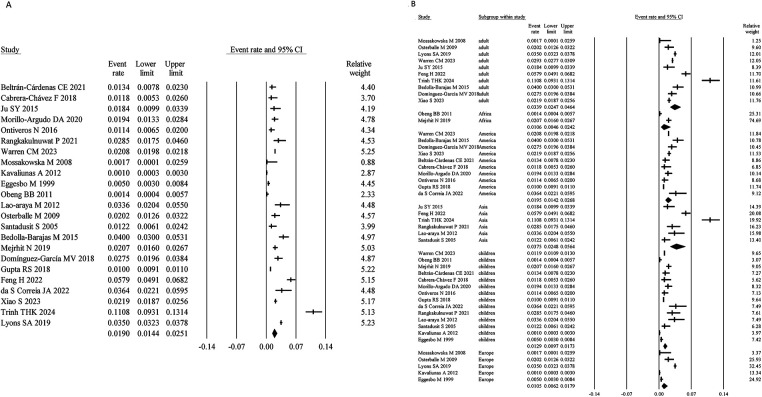
Overall and subgroup prevalence of self-reported symptomatic SA. Panel **(A)** shows the overall prevalence of self-reported symptomatic SA across all populations. Panel **(B)** presents a subgroup analysis of the prevalence by different populations (children and adults) and regions (Asia, America, Europe, and Africa).

Among the included studies, 23 assessed SA using self-reported symptoms, with a total sample size of 167,835. The meta-analysis yielded a pooled prevalence of SA of 1.90% (95% CI: 1.44%–2.51%) (Figure 2A). Heterogeneity analysis indicated a high level of heterogeneity among the studies (I² = 97.67%, *P* < 0.05), suggesting considerable variability in prevalence estimates. Consequently, a random-effects model was applied for the meta-analysis.

Age-stratified analysis showed that the prevalence of self-reported symptomatic SA was 1.29% (95% CI: 0.97%–1.73%) in children, lower than that in adults (3.39%; 95% CI: 2.47%–4.64%). Region-specific analysis revealed prevalence rates of 3.75% (95% CI: 2.48%–5.64%) in Asia, 1.05% (95% CI: 0.62%–1.79%) in Europe, 1.95% (95% CI: 1.42%–2.68%) in America, and 1.06% (95% CI: 0.46%–2.42%) in Africa (Figure 2B).

Egger's regression test showed no evidence of publication bias (*P* = 0.92)); see Supplementary Figure S1 for the funnel plot.

### 3.3 Prevalence of self-reported physician-diagnosed SA

**Figure 3:**
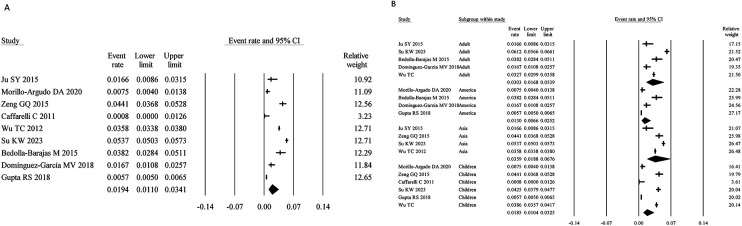
Overall and subgroup prevalence of self-reported physician-diagnosed SA. Panel **(A)** shows the overall prevalence of self-reported physician-diagnosed SA across all populations. Panel **(B)** presents a subgroup analysis of the prevalence by different populations (children and adults) and regions (Asia and America).

A total of 9 studies assessed SA based on self-reported physician diagnosis, with a combined sample size of 51,264. The meta-analysis yielded a pooled prevalence of 1.94% (95% CI: 1.10%–3.41%) (Figure 3A). The heterogeneity analysis indicated significant heterogeneity (I² = 99.21%, *P* < 0.05), necessitating the use of a random-effects model.

Age-stratified analysis revealed that the prevalence in children was 1.85% (95% CI: 1.04%–3.25%), lower than that in adults (3.03%; 95% CI: 1.68%–5.39%). Regional analysis showed prevalence rates of 3.39% (95% CI: 1.88%–6.76%) in Asia and 1.30% (95% CI: 0.62%–2.67%) in America (Figure 3B).

Egger's regression test showed no evidence of publication bias (*P* = 0.36); see Supplementary Figure S2 for the funnel plot.

### 3.4 Prevalence of SPT- or sIgE-based SA

**Figure 4:**
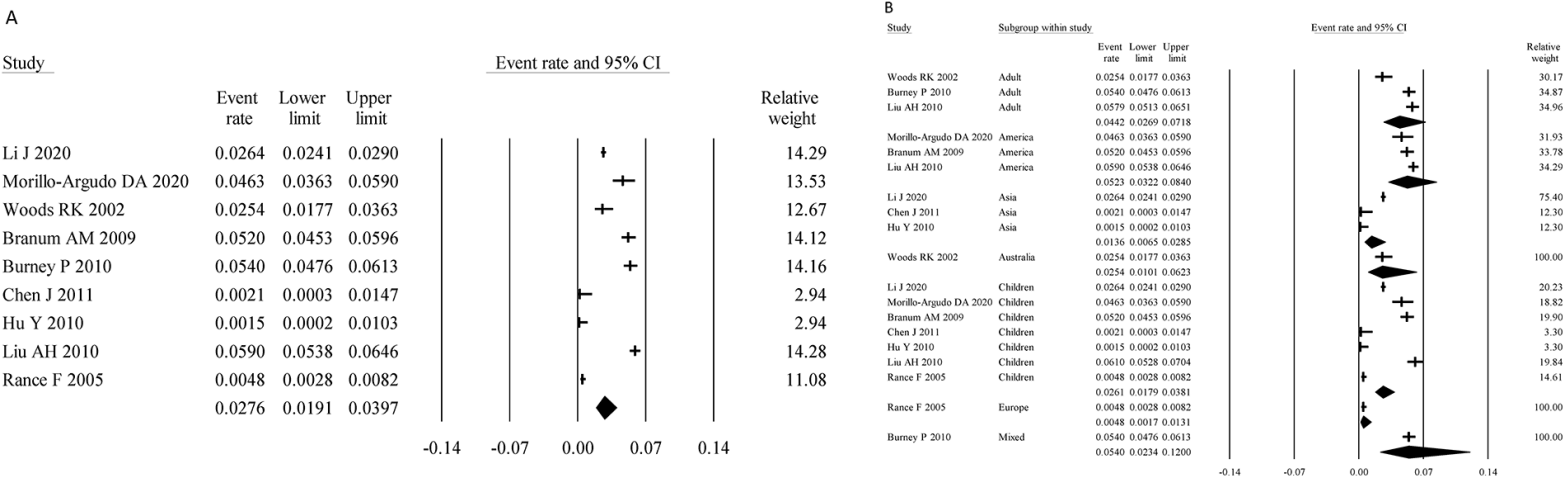
Overall and subgroup prevalence of SPT- or sIgE-based SA. Panel **(A)** shows the overall prevalence of SPT- or sIgE-based SA across all populations. Panel **(B)** presents a subgroup analysis of the prevalence by different populations (children and adults) and regions (Asia and America).

Nine studies assessed SA based on SPT- or sIgE-testing, with a total sample size of 38,459. The pooled prevalence was 2.76% (95% CI: 1.91%–3.97%) (Figure 4A). Heterogeneity analysis revealed high variability (I² = 97.01%, *P* < 0.05), necessitating the use of a random-effects model.

Age-stratified analysis showed a prevalence of 2.61% (95% CI: 1.79%–3.81%) in children, lower than that in adults (4.42%; 95% CI: 2.69%–7.18%). Regional analysis revealed prevalence rates of 1.36% (95% CI: 0.65%–2.85%) in Asia and 5.23% (95% CI: 3.22%–8.40%) in America (Figure 4B).

Egger's regression test showed no evidence of publication bias (*P* = 0.29); see Supplementary Figure S3 for the funnel plot.

### 3.5 Prevalence of SPT- or sIgE-based symptomatic SA

**Figure 5:**
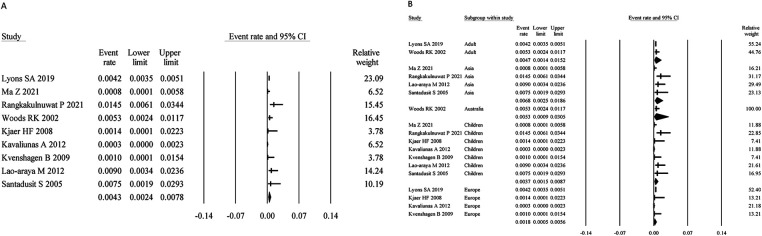
Overall and subgroup prevalence of SPT- or sIgE-based symptomatic SA. Panel **(A)** shows the overall prevalence of SPT- or sIgE-based symptomatic SA across all populations. Panel **(B)** presents a subgroup analysis of the prevalence by different populations (children and adults) and regions (Asia and Europe).

Nine studies assessed SA using SPT- or sIgE-testing combined with symptoms, with a total sample size of 30,779. The pooled prevalence was 0.43% (95% CI: 0.24%–0.78%) (Figure 5A). Heterogeneity analysis showed moderate variability (I² = 62.86%, *P* < 0.05), necessitating the use of a random-effects model.

Age-stratified analysis revealed that the prevalence in children was 0.37% (95% CI: 0.15%–0.87%), slightly lower than that in adults (0.47%; 95% CI: 0.14%–1.52%). Regionally, the prevalence was 0.68% (95% CI: 0.25%–1.86%) in Asia and 0.18% (95% CI: 0.05%–0.56%) in Europe (Figure 5B).

Egger's regression test showed no evidence of publication bias (*P* = 0.71); see Supplementary Figure S4 for the funnel plot.

### 3.6 Prevalence of SA confirmed by food challenge

**Figure 6:**
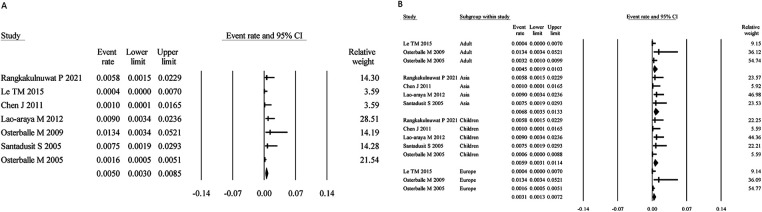
Overall and subgroup prevalence of confirmed by food challenge SA. Panel **(A)** shows the overall prevalence of confirmed by food challenge SA across all populations. Panel **(B)** presents a subgroup analysis of the prevalence by different populations (children and adults) and regions (Asia and Europe).

Seven studies assessed SA using food challenge testing, with a total sample size of 4,652. The meta-analysis yielded a pooled prevalence of 0.50% (95% CI: 0.30%–0.85%) (Figure 6A). Heterogeneity analysis indicated moderate variability (I² = 48.49%, *P* > 0.05), justifying the use of a fixed-effects model.

Age-stratified analysis showed that the prevalence in children was 0.59% (95% CI: 0.31%–1.14%), slightly higher than that in adults (0.45%; 95% CI: 0.19%–1.03%). Regional analysis indicated a prevalence of 0.68% (95% CI: 0.35%–1.33%) in Asia and 0.31% (95% CI: 0.13%–0.72%) in Europe (Figure 6B).

Egger's regression test showed no evidence of publication bias (*P* = 0.25); see Supplementary Figure S5 for the funnel plot.

## 4 Discussion

This meta-analysis systematically evaluated the global prevalence of SA by incorporating 40 studies from various regions and across different age groups. The pooled prevalence estimates were as follows: self-reported symptomatic SA 1.90%, self-reported physician-diagnosed SA 1.94%, SPT- or sIgE-based SA 2.76%, and SPT- or sIgE-based SA combined with clinical symptoms 0.43%, with a food challenge-confirmed SA of 0.50%. This highlights the risk of overestimation when relying solely on self-reported data.

A large cross-sectional study conducted by Helen T. Wang *et al*. during 2015–2016 at the National Opinion Research Center (NORC) at the University of Chicago assessed food allergy prevalence via telephone and online surveys. Their results indicated an overall crustacean allergy prevalence of 1.2% (95% CI: 1.0%–1.3%), which is highly consistent with the self-reported SA prevalence of 1.29% (95% CI: 0.97%–1.73%) observed among children in our study (43). Moreover, our finding of a higher prevalence in adults compared to children aligns with trends observed in mollusk allergies, potentially due to cumulative sensitization with age. Similarly, a meta-analysis by Feng H *et al*. on the global prevalence of food allergies and associated factors reached the conclusion that adult mollusk allergy prevalence was higher than that in children (44). Our study further confirmed significant regional variations in SA prevalence, suggesting an influence of dietary habits, genetic predisposition, and environmental factors (45). Moonesinghe *et al*. published a systematic review in 2016 on the prevalence of shellfish allergy, which included studies published up to 2012 (46). Our meta-analysis updates this by incorporating studies through 2025 and expanding coverage to underrepresented regions such as Asia, Africa, and South America. Moonesinghe's review reported self-reported SA prevalence ranging from 0.1%–5.5% in various populations, with clinically diagnosed prevalence in Taiwan at 3.3% for adults and up to 4.0% for children. Compared to previous estimates, our pooled results suggest that SA prevalence has slightly increased or remained stable in some high-income countries, while it may still be underestimated in several low- and middle-income countries due to limited data or study quality. Additionally, our refined subgroup analyses by diagnostic method and age provide a more comprehensive epidemiological overview.

In our analysis, most prevalence estimates exhibited considerable heterogeneity (I² > 90%), which may be attributed to several factors including geographic and cultural differences, racial and genetic factors, and methodological heterogeneity; for instance, shrimp consumption varies by region, so the higher prevalence observed in Asian regions might be related to the widespread consumption of shrimp and increased exposure to environmental risk factors, whereas lower prevalence rates in Europe and Africa could be due to differing dietary habits, and in coastal areas where seafood consumption is higher, the prevalence of SA may also be elevated, although there is currently a lack of direct literature evidence to support these hypotheses; additionally, variations in genetic susceptibility among different ethnic groups may influence the development of allergies, while differences in sample sizes, study designs (e.g., cross-sectional vs. cohort studies), and data collection methods (e.g., questionnaire surveys vs. clinical testing) across studies may affect comparability and further contribute to the observed heterogeneity. Moreover, the use of varied diagnostic methods—ranging from self-reports to oral food challenges—has introduced further heterogeneity. To better account for this, we categorized the included studies into five diagnostic groups: 1. self-reported symptomatic SA, 2. self-reported physician-diagnosed SA, 3. positive SPT or sIgE SA, 4. probable SA (defined as sensitization plus clinical symptoms), and 5. food challenge-confirmed SA. This classification reflects the current heterogeneity in the literature and enabled structured subgroup analyses in our meta-analysis.

While self-reported SA is widely used in large-scale epidemiological studies due to its convenience, it often overestimates prevalence owing to poor specificity. In contrast, SPT and sIgE indicate sensitization, but not necessarily clinical allergy. The “probable SA” category attempts to bridge this gap and may better reflect true disease burden. Oral food challenge, although the gold standard, is rarely feasible in large-scale studies due to cost, time, and ethical constraints. These diagnostic methods are widely used across existing studies, and almost all prevalence research on SA has employed one or more of these definitions. Thus, adopting this classification scheme provided a practical and comprehensive framework for synthesis.

Understanding SA prevalence is crucial for risk assessment and public health strategies. The discrepancy between self-reported and confirmed allergy prevalence suggests a need for improved diagnostic accuracy in clinical practice. Public health strategies should focus on: Increasing awareness of SA and proper diagnostic methods. Implementing educational programs to prevent unnecessary dietary restrictions. Ensuring access to epinephrine auto-injectors in high-risk populations.

While we followed the PRISMA guidelines closely and employed a comprehensive search strategy encompassing diverse populations, several limitations remain. First, the considerable heterogeneity among studies limits the broad applicability of the pooled prevalence estimates. Second, the paucity of studies from certain regions, particularly in Europe and Africa, may restrict the global generalizability of our findings. Third, differences in the methodological quality of the included studies could affect the reliability of the data and, consequently, the accuracy of the pooled estimates. To address this concern, we categorized studies based on methodological quality and conducted subgroup analyses accordingly. However, the differences in prevalence estimates between high- and lower-quality studies were minimal. Fourth, the failure to differentiate between IgE-mediated and non-IgE-mediated food allergies, as well as food intolerances, might result in an overestimation of the prevalence of IgE-mediated SA. In the studies included in our meta-analysis, the reporting of this information was inconsistent. A substantial number of studies did not specify whether the reported shellfish allergy was IgE-mediated, which limited our ability to conduct stratified analyses. Fifth, to estimate the true prevalence of SA, we adjusted for participant dropout in some studies based on the proportion of individuals who completed the study. However, this proportional adjustment may not fully reflect reality; for example, individuals with severe allergies are more likely to decline participation in oral food challenge (OFC) tests, leading to their underrepresentation in the study samples and potentially biasing the pooled prevalence estimates.

To further enhance our understanding of SA prevalence and its determinants, future research should focus on prospective cohort studies to follow the natural history of SA from childhood into adulthood. Additionally, developing standardized diagnostic criteria is essential to improve comparability across studies and reduce heterogeneity. In high-prevalence regions, further investigation into the genetic and environmental factors contributing to SA is warranted, along with assessments of the impact on quality of life, economic burden, and healthcare resource use. Such efforts will yield a more robust evidence base for public health policies and clinical strategies.

This meta-analysis provides the most comprehensive synthesis to date on the global prevalence of SA, revealing substantial variations by age and region. This underscores the need for standardized diagnostic criteria to improve study comparability and diagnostic accuracy. Future research should focus on optimizing diagnostic protocols and identifying risk factors contributing to SA prevalence.

## Data Availability

Publicly available datasets were analyzed in this study. This data can be found here: All data analyzed in this study were obtained from publicly available sources, including published literature from PubMed, Web of Science, and Embase. The dataset supporting the findings of this study is available from the corresponding author upon reasonable request.
