# Supplementary figures and images for: Prevalence of shrimp allergy: a meta-analysis based on different diagnostic methods
Source: Front Allergy. 2025 Sep 1;6:1635274. doi: 10.3389/falgy.2025.1635274 (PMC12434110; doi:10.3389/falgy.2025.1635274)

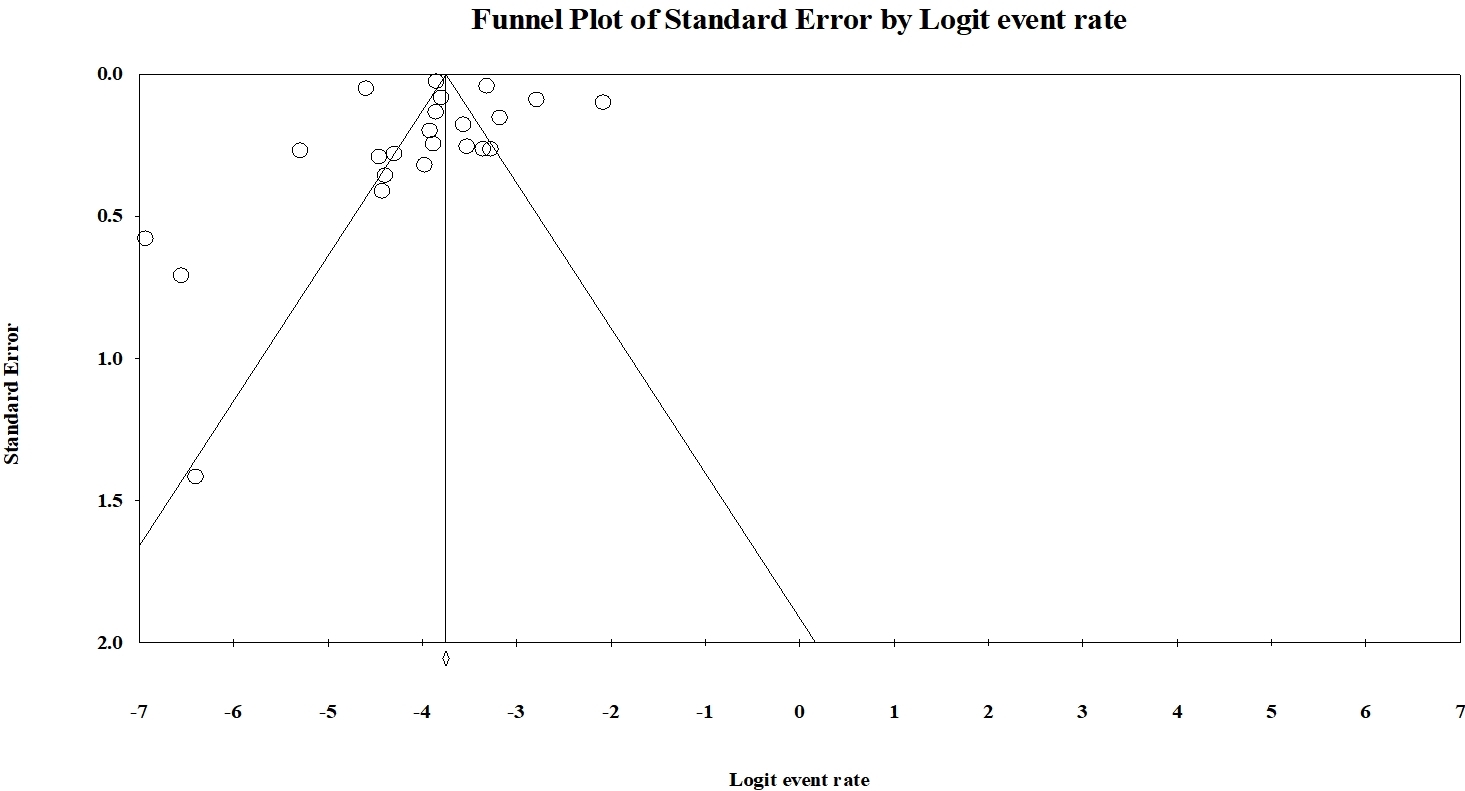

Supplement: Supplementary file 1 [file Image1.jpeg]

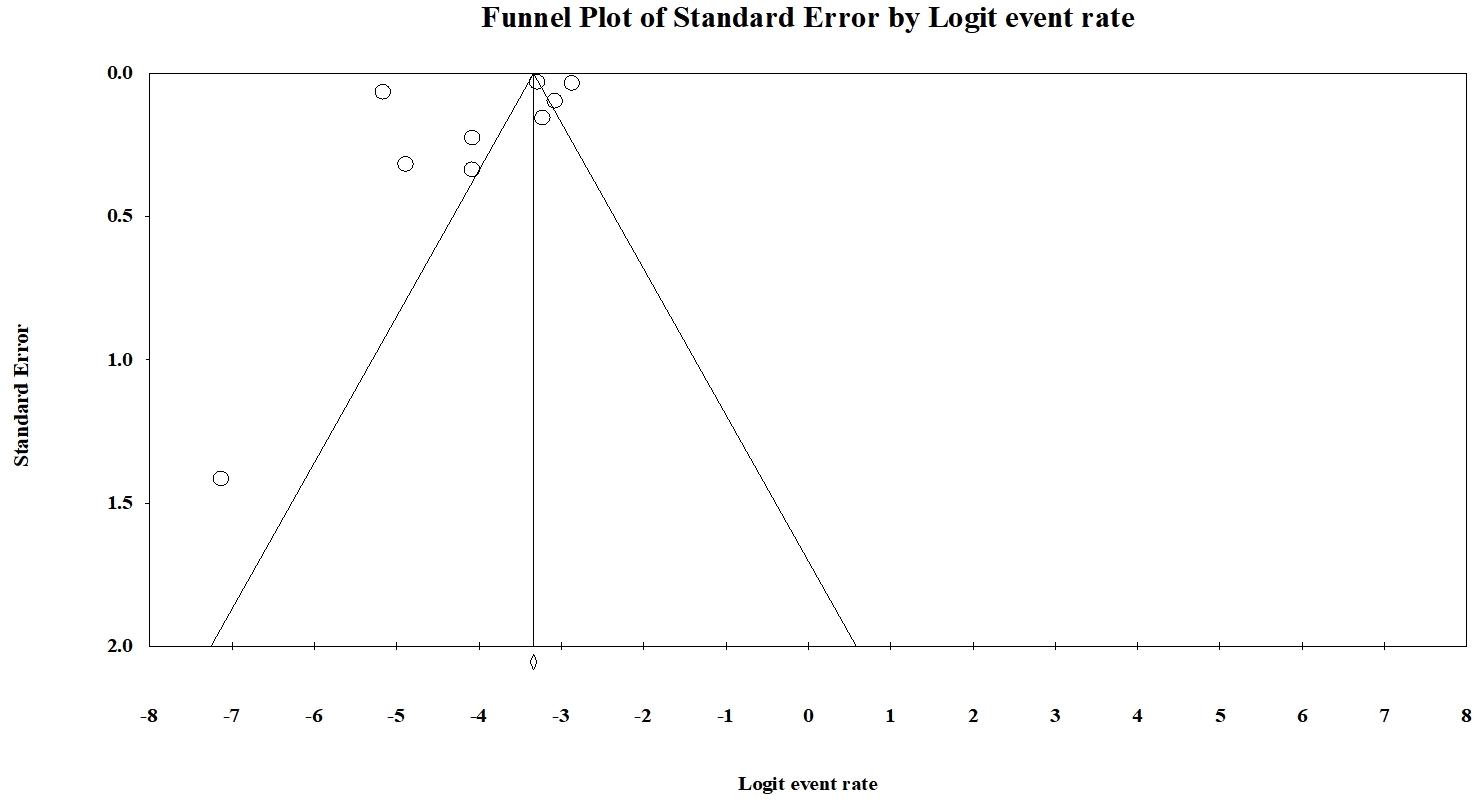

Supplement: Supplementary file 2 [file Image2.jpeg]

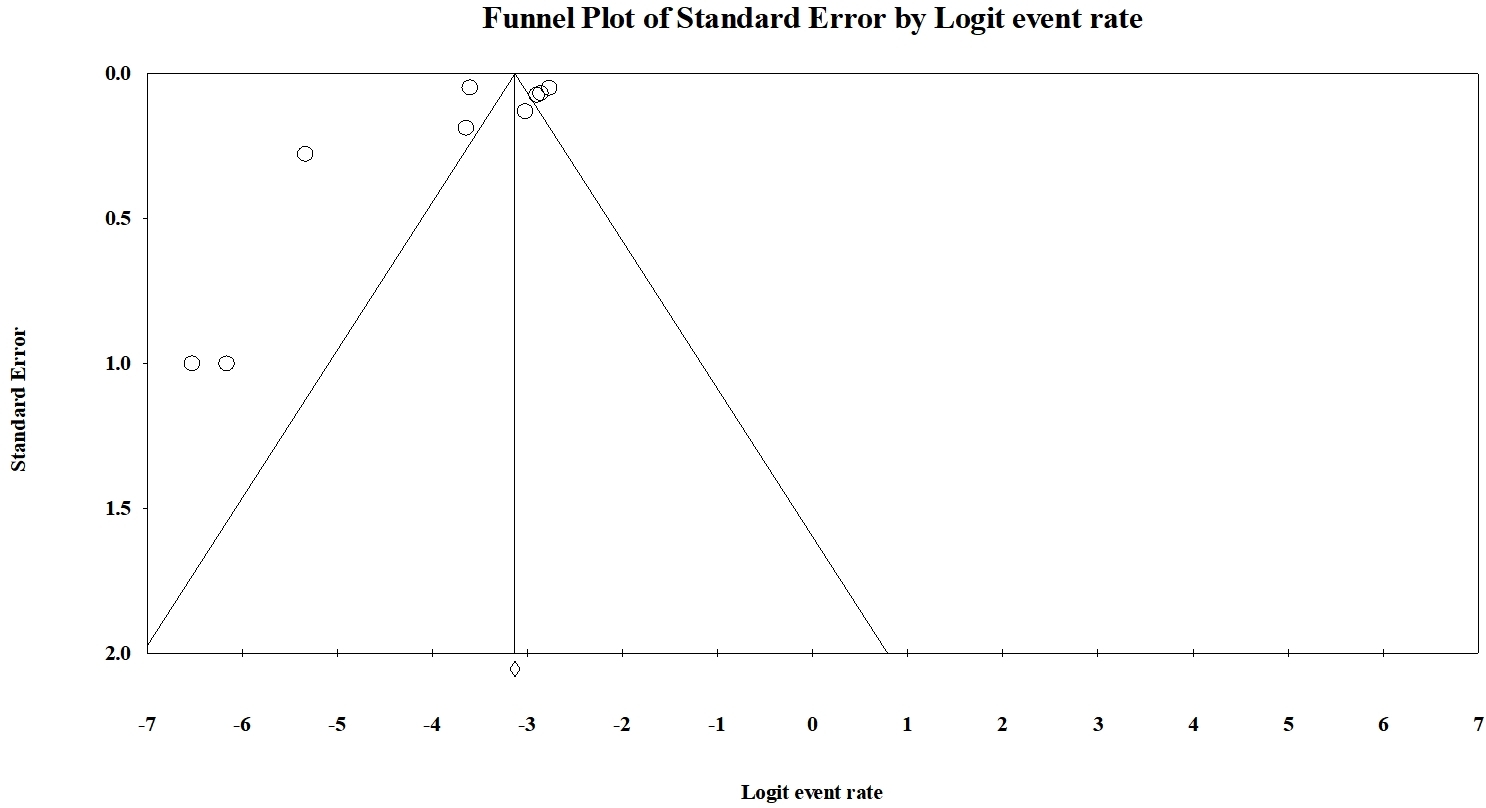

Supplement: Supplementary file 3 [file Image3.jpeg]

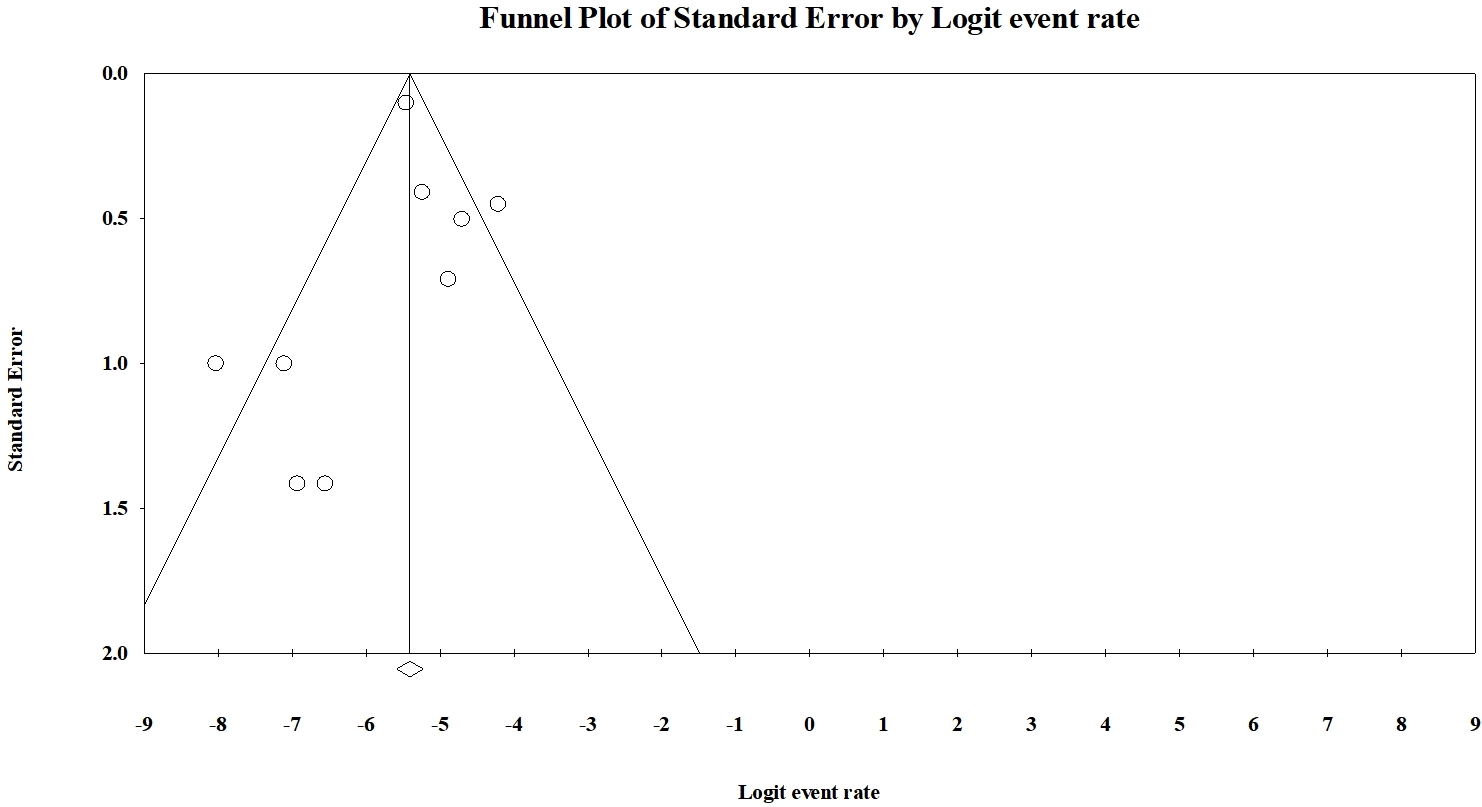

Supplement: Supplementary file 4 [file Image4.jpeg]

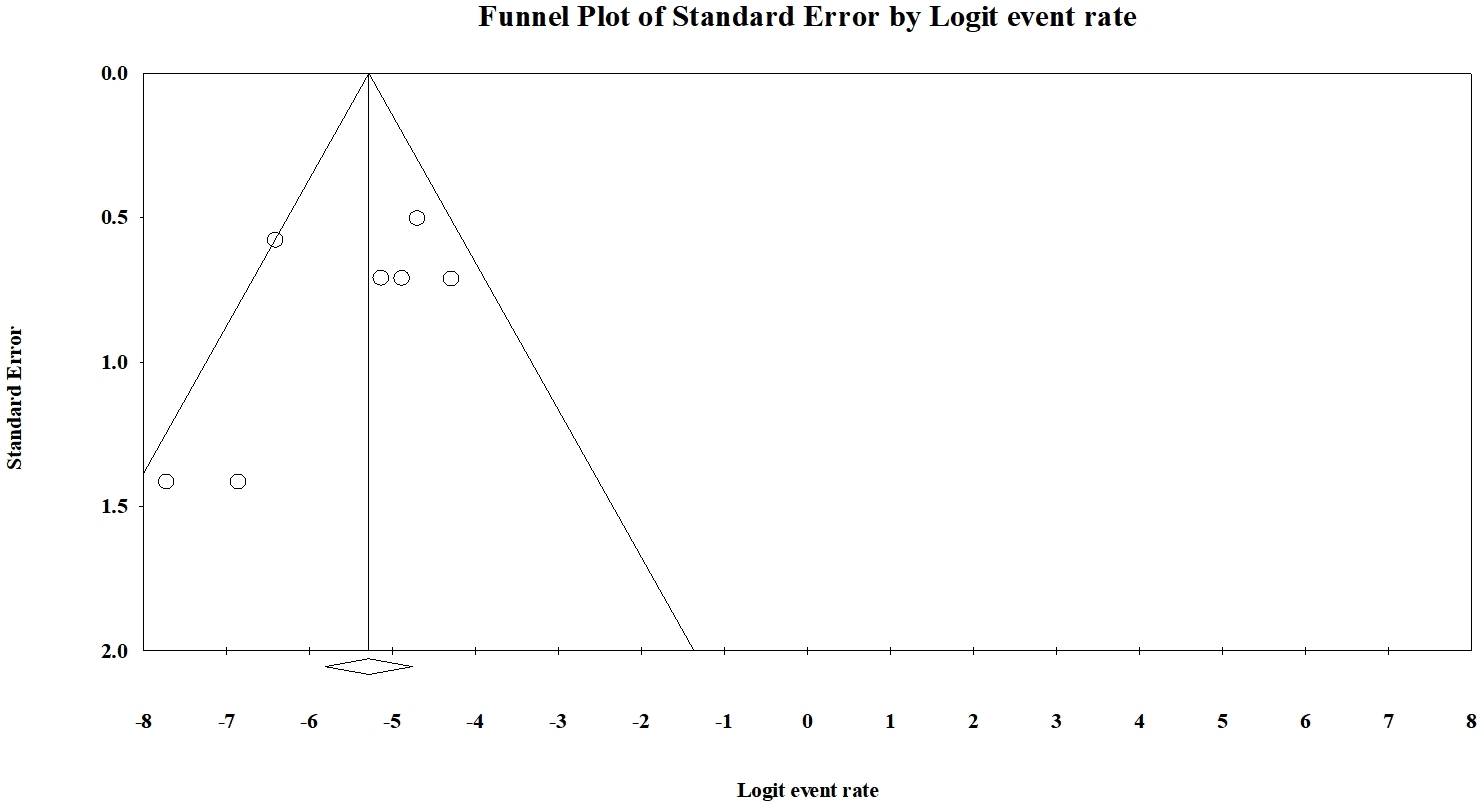

Supplement: Supplementary file 5 [file Image5.jpeg]
