# Supplementary material for: Prevalence of shrimp allergy: a meta-analysis based on different diagnostic methods
Source: Front Allergy. 2025 Sep 1;6:1635274. doi: 10.3389/falgy.2025.1635274 (PMC12434110; doi:10.3389/falgy.2025.1635274)
Supplement: Supplementary file 6 [file Table1.docx]

**Supplementary Figure Legends**

Figure S1. Funnel plot of standard error by logit event rate for the prevalence of self-reported symptomatic SA.

Figure S2. Funnel plot of standard error by logit event rate for the prevalence of self-reported Physician-diagnosed SA.

Figure S3. Funnel plot of standard error by logit event rate for the prevalence of SPT- or sIgE-based SA.

Figure S4. Funnel plot of standard error by logit event rate for the prevalence of SPT- or sIgE-based Symptomatic SA.

Figure S5. Funnel plot of standard error by logit event rate for the prevalence of SA Confirmed by Food Challenge.
